# Awareness and understanding of cytomegalovirus infection among polish pregnant women: A CASP-W cross-sectional study

**DOI:** 10.1016/j.pmedr.2025.103348

**Published:** 2025-12-11

**Authors:** Magda Rybak-Krzyszkowska, Michał Strus, Hubert Huras, Wojciech Górczewski, Maciej W. Socha, Lidia Stopyra, Dorota Sys

**Affiliations:** a Department of Obstetrics and Perinatology, University Hospital, Kraków, Poland; b Hi-Gen Centrum Medyczne, Kraków, Poland; c Obstetrics and Perinatology Department, Medical Colleage of Jagiellonian University, Cracov, Poland; d Independent Public Health Care Facility "Bl. Marta Wiecka County Hospital", Bochnia, Poland; e Department of Perinatology, Gynecology and Gynecologic Oncology, Collegium Medicum in Bydgoszcz, Nicolaus Copernicus University, 85-821 Bydgoszcz, Poland; f Department of Infectious and Tropical Diseases, Andrzej Frycz Modrzewski Krakow University Medical College, 30-705 Kraków, Poland; g Department of Translational Immunology and Experimental Intensive Therapy, Centre of Postgraduate Medical Education, Warsaw, Poland

**Keywords:** Cytomegalovirus infections, Pregnancy, Health knowledge, attitudes, practice, Cross-sectional studies, Surveys and questionnaires, Socioeconomic factors, Health education

## Abstract

Objectives: To evaluate awareness and understanding of congenital cytomegalovirus infection among pregnant women in Poland and to identify factors associated with higher knowledge.

Methods: A web-based cross-sectional study was conducted in Poland between February and July 2024, using an anonymous online questionnaire distributed via obstetric–gynecologic clinics and social media. A total of 1015 fully completed responses were analyzed. Knowledge was assessed using a 15-item score and dichotomized at the median. Group comparisons were performed using *t*-tests or Wilcoxon tests, and univariate and multivariate logistic regression models were used to identify factors associated with knowledge (*p* < 0.05).

Results: The mean knowledge score was 7.70 ± 3.60, with no difference between primiparous and multiparous women. Higher odds of high knowledge were associated with daily contact with children under three years of age (OR 1.60; 95 %CI 1.20,2.14) and comfortable household income (OR 1.56; 95 %CI 1.20,2.04). Lower knowledge was associated with public prenatal care (OR 0.65; 95 %CI 0.46,0.94), residence in medium-sized cities (OR 0.59; 95 %CI 0.39,0.88), and rural areas (OR 0.61; 95 %CI 0.44,0.85).

Conclusions: Pregnant women in Poland demonstrate only moderate awareness of congenital cytomegalovirus, with significant socioeconomic and healthcare-related disparities, indicating a need for early targeted education within public antenatal services.

## Highlights

• First national survey of cytomegalovirus awareness among 1015 Polish pregnant women.
• Mean cytomegalovirus knowledge score was moderate and independent of parity.
• Higher knowledge associated with private care, higher income, and child contact.
• Public prenatal care and rural or mid-sized city residence predicted lower knowledge.
• Findings support targeted cytomegalovirus education in public antenatal services.

## 1 Introduction

Cytomegalovirus (CMV), a double-stranded DNA virus from the herpesvirus family, establishes lifelong latency and may reactivate during immunosuppression. Global seroprevalence ranges from 52 % to 97 %, underscoring its ubiquity. (Cito et al., 2020; Work et al., 2022) Although often asymptomatic, CMV is the leading infectious cause of developmental disabilities, including hearing loss and neurodevelopmental impairment in children. (Weimer et al., 2020)

Vertical transmission during pregnancy is the primary route of concern, especially in utero infection during primary maternal exposure, which carries a transmission risk of up to 30 % in the first trimester. (Donalisio et al., 2020; Davis et al., 2017) Non-primary infections pose lower riskstransmission rates below 2 %. (Chiopris et al., 2020) Adult CMV acquisition occurs via sexual contact, respiratory droplets, transplantation, and, less frequently, transfusions. (Menakaya, 2021; Solomon and Mastropietro, 2013)

Congenital CMV affects 1 %–5 % of births in developing countries, and remains the most common intrauterine viral infection. (Chiopris et al., 2020; Manicklal et al., 2013) Clinical sequelae can include microcephaly and cystic lesions, periventricular calcifications, hepatosplenomegaly, and sensorineural hearing loss. (Slots and Slots, 2000; Neubauer et al., 2025; Picone et al., 2013) While only 10–15 % of infected neonates are symptomatic at birth, many asymptomatic cases later develop impairments such as learning difficulties or hearing deficits. (Wade et al., 2024; Lazzarotto et al., 2020; Lindholm and O'Keefe, 2019) Ongoing monitoring is therefore critical. (Hughes and Gyamfi-Bannerman, 2016) In Poland, routine cytomegalovirus screening is not included in the national antenatal care program, and preventive strategies focus primarily on hygiene education and risk awareness among pregnant women, in line with current national recommendations. Tests for CMV are performed with the Patient's co-financing. (*Obwieszczenie Ministra Zdrowia z dnia 9 czerwca 2023 r. w sprawie ogłoszenia jednolitego tekstu rozporządzenia Ministra Zdrowia w sprawie standardu organizacyjnego opieki okołoporodowej [Internet]. [cited* 2025 Oct 22], 2025; Rybak-Krzyszkowska et al., 2025)

The awareness of maternal health regarding CMV infection is crucial in preventing fetal complications and ensuring better health outcomes for newborns. Current literature emphasizes the significance of raising awareness among pregnant women about the potential risks associated with CMV infection. (Greye et al., 2022; Liberati et al., 2024) Maternal understanding of CMV transmission routes—primarily through bodily fluids such as saliva and urine—enables women to adopt preventive hygiene practices that can substantially reduce the risk of acquiring the virus during pregnancy. (Barber et al., 2020; Billette de Villemeur et al., 2020)

Despite the clinical importance of CMV, many pregnant women remain unaware of the infection and its risks. In Germany, 60 % of surveyed pregnant women lacked knowledge about CMV-related threats. (Greye et al., 2022) Qualitative data further show that women often feel poorly informed by healthcare providers, missing key opportunities for prevention. (Barber et al., 2020) This highlights systemic shortcomings in CMV educationpotentially limiting the uptake of protective behaviors and contributing to the persistent burden of congenital CMV.

The primary aim of this web-based cross-sectional study was to assess the awareness and understanding of CMV infection among pregnant women. Additionally, we sought to identify demographic and socio-economic factors associated with varying levels of knowledge about CMV. The findings from this study will inform tailored educational strategies, facilitating improved maternal education and ultimately enhancing maternal and neonatal health outcomes.

## 2 Material and method

### 2.1 Study design and population

This web-based cross-sectional study was conducted between February 19, 2024, and July 3, 2024, using an anonymous online questionnaire developed in Microsoft Office Forms. The web-based format was chosen to maximize accessibility, geographical reach, and participant anonymity, enabling efficient recruitment across different regions of Poland. The study constituted the patient-reported part of the CASP – CMV Awareness Study in Poland. The questionnaire was developed and analyzed according to the Knowledge, Attitudes, and Practices (KAP) framework, which distinguishes between awareness (exposure or recognition), knowledge (factual understanding), and understanding (interpretative application of knowledge). (*Advocacy, communication and social mobilization for TB control: a guide to developing knowledge, attitude and practice surveys. Geneva: World Health Organization: Stop TB Partnership*, 2008) The manuscript was prepared following the Strengthening the Reporting of Observational Studies in Epidemiology (STROBE) guidelines. (von et al., 2007)

Participants were recruited through healthcare professionals, including obstetricians-gynecologists and midwives, who distributed informational leaflets and posters with QR codes in outpatient clinics. Additionally, professionals disseminated information about the study through their social media platforms.

Pregnant women aged 18 years or older, residing in Poland, fluent in Polish, and with internet access through any device (computer, laptop, smartphone, or tablet) were eligible for participation. Women at any gestational age were invited. Participation was voluntary, and informed consent was considered granted upon completing the questionnaire. In total, 1061 women completed the questionnaire. After excluding incomplete responses, 1015 fully completed surveys were included in the final analysis. The completion rate was 95.66 % (1015/1061), defined as the proportion of fully completed questionnaires among all surveys that were started (denominator: all respondents who began the questionnaire). Because recruitment was decentralized (QR codes/posters and social media), the total number of women initially reached (impressions) could not be reliably ascertained; thus, the completion rate is calculated relative to survey starts, not invitations or page views.

### 2.2 Measures

The questionnaire assessed participants' awareness of CMV infection and other infectious diseases, perceptions of CMV as a health concern, knowledge regarding transmission, symptoms, preventive measures, screening, and treatment. It also collected information on sources and timing of knowledge acquisition, willingness to undergo CMV screening, factors influencing decision-making, as well as sociodemographic characteristics such as age, educational attainment, place of residence, household income, marital status, parity, gestational age, pregnancy planning, type of prenatal care, and caregiving or professional engagement with young children. A knowledge score was calculated for each respondent by assigning one point for each correct answer. The scores were then summed to generate a total knowledge score. Based on the median score of eight points, participants were categorized into two groups: low knowledge (score < 8) and high knowledge (score ≥ 8).

The questionnaire was developed based on a comprehensive literature review and reviewed by a multidisciplinary team of experts, including obstetricians-gynecologists, infectious disease specialists, and health science researchers. A pilot study involving 15 pregnant women was conducted to verify the clarity and comprehensibility of the questionnaire. Participants were encouraged to provide feedback regarding any difficulties in understanding the questions. The research team analyzed the collected comments, and appropriate modifications were incorporated before the main data collection phase.

To minimize potential bias, only fully complete questionnaires were included in the analysis. Participation was entirely anonymous, and no incentives were offered that could affect respondents' answers.

The minimum required sample size was calculated based on the number of pregnancies registered in Poland in 2023, according to the Polish Central Statistical Office (GUS 2024) data, which equals 272,451. Assuming a 5 % margin of error and an 80 % confidence level, a minimum of 384 participants was determined as necessary for adequate statistical power.

### 2.3 Statistical analysis

Comparisons between groups were performed using Student's *t*-test for normally distributed variables and the Wilcoxon rank-sum test for non-normally distributed variables. Univariate logistic regression models first examined associations between independent variables and the level of knowledge. Variables significantly associated with knowledge level were subsequently included in a multivariate logistic regression model. A *p*-value of less than 0.05 was considered statistically significant. All statistical analyses were conducted using R Studio software with the R programming language.

### 2.4 Ethical considerations

The study was conducted following the ethical principles outlined in the Declaration of Helsinki. Only anonymized data were collected and analyzed. According to applicable Polish legislation, the study did not meet the definition of a medical experiment under Article 21 of the Act on the Profession of Physician (December 5, 1996) nor that of a clinical trial as defined by Regulation 536/2014 and the Act on Clinical Trials of Medicinal Products Used in Humans (March 9, 2023). Therefore, obtaining ethical committee approval was not required.

## 3 Results

A total of 1015 pregnant women were included in the final analysis, comprising 593 primiparous women and 422 multiparous women. Multiparous women were significantly older than primiparous women (mean age 31.8 ± 3.5 years vs. 30.1 ± 3.8 years, *p* < 0.01). Primiparous women more often lived in large and very large cities (42.16 % vs. 34.60 % in very large cities, *p* < 0.01), whereas multiparous women more frequently resided in small towns and rural areas (25.59 % and 29.15 %, respectively). A higher percentage of primiparous women had attained higher education compared to multiparous women (87.02 % vs. 77.96 %, *p* < 0.01). Marriage was more common among multiparous women than among primiparous women (91.00 % vs. 83.31 %, *p* < 0.01). Unplanned pregnancies were significantly more frequent among multiparous women (15.64 % vs. 7.42 %, *p* < 0.01). Multiparous women were also more likely to have children under the age of five (78.91 % vs. 0 %, p < 0.01) and to care for or work with children under the age of three (47.16 % vs. 12.82 %, p < 0.01). (Table 1).

**Table 1:** Sociodemographic and obstetric characteristics of primiparous and multiparous women in Poland, February–July 2024 (*n* = 1015).

	**Primiparous (*n* = 593)** mean ± SD n (%)	**Multiparous (*n* = 422)** mean ± SD n (%)	**p-value**
Age	30.1 ± 3.8	31.8 ± 3.5	<0.01
Gestational week	22.6 ± 9.7	23.6 ± 10.6	0.12
Week of pregnancy recognition	5.0 ± 3.0	5.1 ± 2.7	0.2
Planned pregnancy	549 (92.58)	356 (84.36)	<0.01
Primary prenatal care: private	503 (84.82)	359 (85.07)	0.95
Having children under 5 years	0 (0.00)	333 (78.91)	<0.01
Caring for/working with children under 3 years	76 (12.82)	199 (47.16)	<0.01
Married	494 (83.31)	384 (91.00)	<0.01
Residence: very large city (>100,000)	250 (42.16)	146 (34.60)	<0.01
Comfortable household income	302 (50.93)	188 (44.55)	0.06
Higher education	516 (87.02)	329 (77.96)	<0.01

NOTE: SD - standard deviation.

Regarding general awareness, no significant differences were found between primiparous and multiparous women in perceiving CMV infection as a serious health concern or in recognizing that CMV can be transmitted from mother to fetus. Similarly, there were no significant differences in the belief that CMV screening is mandatory, awareness of the possibility of preventing fetal infection after maternal infection, or awareness of the possibility of treating a fetus infected with CMV. Both groups also had similar levels of knowledge regarding the impact of CMV on hearing loss in children. (Table 2).

**Table 2:** Awareness and attitudes toward cytomegalovirus among primiparous and multiparous women in Poland, February–July 2024 (*n* = 1015).

	**Primiparous (n = 593)** mean ± SD n (%)	**Multiparous (n = 422)** mean ± SD n (%)	**p-value**
CMV as a serious health problem	410 (69.14)	299 (70.85)	0.24
Knowledge of mother-to-child transmission	467 (78.75)	323 (76.54)	0.55
Knowledge that CMV screening is not mandatory	227 (38.28)	161 (38.15)	0.99
Knowledge that CMV infection is more frequent than genetic syndromes	170 (28.67)	133 (31.52)	0.03
Knowledge of the prevention of CMV fetal infection	222 (37.44)	159 (37.68)	1
Knowledge that the gestational age of infection matters	345 (58.18)	262 (62.09)	0.35
Knowledge about the possibility of fetal treatment	192 (32.38)	140 (33.18)	0.88
Knowledge that CMV may cause hearing loss	315 (53.12)	232 (54.98)	0.84
Willingness to perform CMV screening	535 (90.22)	381 (90.28)	1
Willingness to undergo invasive diagnostics	416 (70.15)	280 (66.35)	0.33

NOTE: CMV – cytomegalovirus; SD - standard deviation.

Multiparous women were significantly more likely to have encountered information about CMV during the third trimester (11.14 % vs. 5.40 %, *p* < 0.01). Sources of information did not significantly differ between groups, although primiparous women slightly more often indicated receiving information through online pregnancy portals, social media, and their healthcare provider, without statistical significance. There were no differences between groups regarding willingness to perform CMV screening if not reimbursed or willingness to undergo invasive prenatal diagnostic procedures if necessary. (Table 3).

**Table 3:** Knowledge about cytomegalovirus, symptoms, transmission routes, prevention, and information sources, among pregnant women in Poland, February–July 2024 (*n* = 1015).

	**Primiparous (n = 593)** mean ± SD n (%)	**Multiparous (n = 422)** mean ± SD n (%)	**p-value**
Knowledge of saliva/urine transmission	353 (59.53)	244 (57.82)	0.63
Knowledge of transmission via raw meat	74 (12.48)	52 (12.32)	1
Knowledge of transmission via cat contact	29 (4.89)	27 (6.40)	0.37
Knowledge of asymptomatic cytomegalovirus infection	435 (73.36)	340 (80.57)	0.01
Identification of fever as a symptom	317 (53.46)	213 (50.47)	0.38
Identification of muscle aches as a symptom	239 (40.30)	170 (40.28)	1
Knowledge that most infected newborns are asymptomatic	259 (43.68)	206 (48.82)	0.12
Knowledge of prevention by handwashing after diaper change	276 (46.54)	205 (48.58)	0.56
Source of information: social media	214 (36.09)	163 (38.63)	0.45
Source of information: pregnancy websites	216 (36.42)	164 (38.86)	0.47
Source of information: healthcare provider	200 (33.73)	125 (29.62)	0.19
Timing of information: 3rd trimester	32 (5.40)	47 (11.14)	<0.01
High overall knowledge level	316 (53.29)	234 (55.45)	0.54
Knowledge test score (mean ± SD)	7.7 ± 3.5	7.8 ± 3.7	0.62

NOTE: SD - standard deviation.

When assessing detailed knowledge about CMV, multiparous women were more likely to know that the incidence of CMV infection is higher than that of common genetic syndromes, such as Down syndrome (31.52 % vs. 28.67 %, *p* = 0.03). Multiparous women more frequently reported being familiar with other important infections and conditions, including group B streptococcus infection (91.71 % vs. 76.05 %, *p* < 0.01), parvovirus B19 infection (79.38 % vs. 62.06 %, p < 0.01), and spinal muscular atrophy (88.39 % vs. 82.12 %, *p* = 0.008). Furthermore, multiparous women were more likely to recognize that CMV infection during pregnancy can be asymptomatic (80.57 % vs. 73.36 %, *p* = 0.01). (Table 3).

Finally, no significant differences were found between the groups in the overall knowledge test scores (mean 7.8 ± 3.7 for multiparous women vs. 7.7 ± 3.5 for primiparous women, *p* = 0.63). The proportion of women classified as having a high knowledge level was similar between the groups (55.45 % among multiparous vs. 53.29 % among primiparous, *p* = 0.54). (Table 3).

To assess factors associated with the level of knowledge about CMV infection, participants' scores were divided based on the median value of eight points, following a data-driven approach commonly applied when no validated cut-off is available.Univariate logistic regression models were constructed to evaluate the association between selected variables and the level of knowledge. Women who primarily used public healthcare services during pregnancy had lower odds of achieving a high knowledge score compared to those using private services (OR = 0.81, SE = 0.04, *p* < 0.01). Caring for or working with children under three years of age was associated with higher knowledge levels (OR = 1.11, SE = 0.04, *p* < 0.01). Place of residence was also influential: living in a large city (population 30,000–99,999) (OR = 0.85, SE = 0.05, p < 0.01), a small town (population 1000–29,999) (OR = 0.92, SE = 0.04, *p* = 0.03), or a rural area (population 〈1000) (OR = 0.86, SE = 0.04, p < 0.01) was associated with lower odds of having high knowledge compared to living in very large cities. In addition, participants who reported living comfortably on their current household income had higher odds of having a high knowledge score (OR = 1.14, SE = 0.03, p < 0.01). (Table 4).

**Table 4:** Univariate logistic regression models for factors associated with high cytomegalovirus knowledge among pregnant women in Poland, February–July 2024 (n = 1015).

	**Odds ratio**	**Standard error**	**p-value**
Planned pregnancy	1,02	0,05	0,6
Primary prenatal care: public	0,87	0,04	<0,01
Number of children	1,02	0,02	0,37
Children under 5 years: yes	1,03	0,03	0,31
Caring for/working with children under 3 years: yes	1,11	0,04	<0,01
Residence: large city (30 k–99 k)	0,85	0,05	<0,01
Residence: small town (1 k–29 k)	0,92	0,04	0,03
Residence: rural area (<1 k)	0,86	0,04	<0,01
Household income: very difficult	0,82	0,19	0,29
Household income: difficult	0,88	0,09	0,14
Household income: comfortable	1,14	0,03	<0,01
Education: secondary	1,46	0,5	0,44
Education: higher	1,78	0,49	0,24
Marital status: single	0,58	0,35	0,12
Marital status: stable relationship	0,94	0,05	0,18
Maternal age	1,00	<0,01	0,32
Gestational week	1,00	<0,01	0,07
Week of pregnancy recognition	1,00	<0,01	0,42

Other variables, such as pregnancy planning status, number of children, educational attainment, marital status, maternal age, gestational week, and week of pregnancy recognition, were not significantly associated with knowledge levels in the univariate models. (Table 4).

**Fig. 1:**
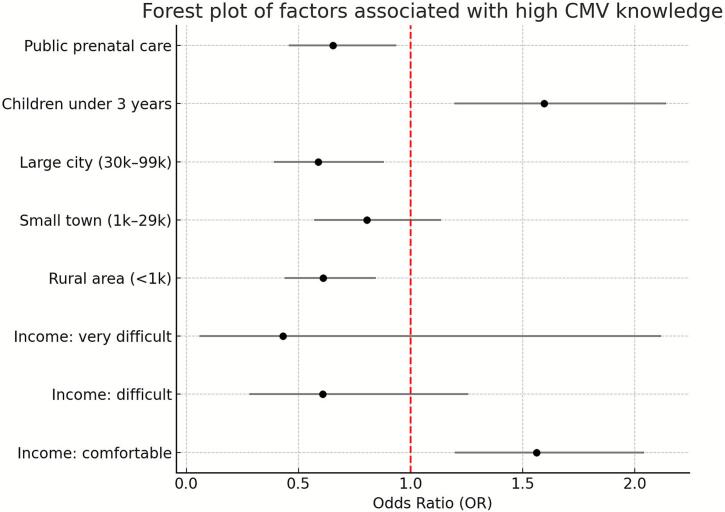
Forest plot presenting factors associated with high cytomegalovirus knowledge among pregnant women in Poland, February–July 2024.

In the next step, a multivariate logistic regression analysis was performed to identify independent factors associated with a higher level of knowledge about CMV infection. Caring for or working with children under the age of three significantly increased the odds of achieving a high knowledge level (OR = 1.6, 95 % CI: 1.12,2.14). Similarly, reporting a comfortable standard of living based on household income was associated with higher knowledge levels compared to those merely managing on their current income (OR = 1.56, 95 % CI: 1.12,2.04). (Table 5, Fig. 1).

**Table 5:** Multivariate logistic regression model assessing factors associated with high cytomegalovirus knowledge among pregnant women in Poland, February–July 2024 (n = 1015).

	**Odds Ratio**	**95 % Confidence Interval**
Primary prenatal care: public	0.65	0.46,0.94
Caring for/working with children under 3 years	1.6	1.2, 2.14
Residence: large city (30,000–99,999)	0.59	0.39, 0.88
Residence: small town (1000–29,999)	0.80	0.57, 1.14
Residence: rural area (〈1000)	0.61	0.44, 0.85
Household income: very difficult	0.43	0.06, 2.12
Household income: difficult	0.61	0.28, 1.26
Household income: comfortable	1.56	1.2, 2.04

Conversely, using primarily public healthcare services during pregnancy was associated with lower odds of achieving a high knowledge level (OR = 0.65, 95 % CI: 0.46,0.94Place of residence also played a significant role. Living in a large city with a population between 30,000 and 99,999 (OR = 0.59, 95 % CI: 0.39,0.88) or in a rural area with fewer than 1000 inhabitants (OR = 0.61, 95 % CI: 0.44,0.85) was associated with lower knowledge levels compared to living in a very large city (population > 100,000). (Table 5, Fig. 1).

## 4 Discussion

This nationwide, web-based cross-sectional study (CASP-W) investigated determinants of pregnant women's knowledge about congenital cytomegalovirus (CMV) in Poland. The findings suggest that CMV knowledge remains suboptimal (mean 7.7/15) and is not significantly influenced by parity. Instead, higher knowledge levels were associated with residence in very large cities, access to private prenatal care, a comfortable household income, and daily contact with young children. These patterns are consistent with prior studies from France, the Netherlands, and Switzerland, which linked higher CMV knowledge to higher education, urban residence, and professional or parental contact with children. (Cordier et al., 2012; Pereboom et al., 2013; Hussami et al., 2025; Willame et al., 2015)

Importantly, our study are consistent withs the view that mere awareness is insufficient for prevention. Similar to results from France and Germany, our participants who had encountered CMV information still demonstrated only partial understanding of transmission routes or consequences. (Greye et al., 2022; Cordier et al., 2012) This “superficial awareness” is well-documented in Swiss, Japanese, and US cohorts, where fewer than 2 % of respondents correctly answered all knowledge questions. (Kobayashi et al., 2021; Suga et al., 2021; Cannon et al., 2012) Consistent with these findings, our data show that women in Poland often encountered CMV information late in pregnancy, particularly among multiparous women, potentially limiting opportunities for primary prevention.

The lack of knowledge improvement with increasing parity contradicts expected results and aligns with findings from studies in Switzerland and Minnesota, which found either weak or no associations. (Pereboom et al., 2013; Hussami et al., 2025) As multiparas in our study more often received CMV-related information only in the third trimester, the timing of counselling appears crucial. These observations mirror earlier concerns raised by Tastad et al., who argued that public health messaging frequently fails to reach pregnant women early enough to alter behavior. (Tastad et al., 2019) The lack of difference in CMV knowledge between primiparous and multiparous women may reflect confounding by urban residence, as primiparous participants were more often city dwellers. When adjusted for place of residence, parity no longer contributed independently to knowledge level. Unlike reports from other European cohorts, our findings indicate that parity does not influence CMV knowledge once urban residence is accounted for, highlighting a distinct pattern within the Polish antenatal context where counselling is often delayed until late pregnancy. In Poland, routine CMV screening is not included in the national antenatal care program, and systematic counselling on congenital CMV prevention is not formally implemented, which may partly explain the limited impact of parity on knowledge levels. Preventive education thus depends largely on individual providers' initiative rather than standardized national guidance. We believe that the Polish guidelines on the management of CMV infection in pregnant women, issued in September of this year, will significantly improve this situation and introduce better care for pregnant patients, including in the educational aspect. (Rybak-Krzyszkowska et al., 2025)

Socioeconomic gradients in knowledge, observed in our study, are similarly described in US and European literature. (Cordier et al., 2012; Willame et al., 2015; Castillo et al., 2022) Polish women who used public care or lived in small towns and rural areas had significantly lower CMV knowledge. This finding suggests that discrepancies are not unique to the Polish healthcare system but may reflect broader structural inequalities within antenatal care provision across high-income countries. (Hussami et al., 2025) However, our online recruitment strategy, which overrepresented highly educated users, may have slightly inflated average scores—just as noted by Kobayashi et al. in Japan and Trzcińska. (Kobayashi et al., 2021; Trzcińska et al., 2017)

Moreover, despite consistent public health recommendations, the link between knowledge and behavioral prevention remains poorly understood. Our results did not include behavioral outcomes, but several authors have called for future studies pairing knowledge with hygiene behaviors or biomarker-confirmed seroconversion to establish causality. (Pereboom et al., 2013; Castillo et al., 2022; Wizman et al., 2016)

The findings highlight the need for a structured, nationally coordinated CMV education program integrated into routine public antenatal care. Early implementation in the first trimester is essential, especially in rural and smaller-town settings, where knowledge gaps were most pronounced. Healthcare professionals should receive a concise, evidence-based counselling guide covering CMV transmission, fetal risks, and hygiene measures. Materials must be accessible (B1–B2 level), visually engaging, and available in both print and digital formats, including for linguistic minorities. Follow-up reminders during pregnancy (e.g., via SMS or mobile apps) can reinforce key messages. This approach aligns with previous research demonstrating the effectiveness of brief, targeted education in improving maternal knowledge and preventive intentions. (Cordier et al., 2012; Hussami et al., 2025; Willame et al., 2015; Castillo et al., 2022) Basic monitoring tools, such as structured knowledge checks and documentation of counselling, would support evaluation and scaling. Integrating CMV prevention into national antenatal guidelines and professional training curricula could further strengthen policy alignment and ensure consistent implementation across healthcare settings.

Although CASP-W included over a thousand respondents from across Poland, several limitations must be considered. The survey was distributed via pregnancy-related websites and social media, which may have attracted women more engaged with health information, leading to inflated knowledge scores. Similar selection bias has been reported in other online CMV studies. (Hussami et al., 2025; Kobayashi et al., 2021) National data indicate that 75 % of pregnant women in Poland utilize these resources; however, our respondents had a higher proportion of tertiary education, at 90 %, compared to 58 % of Polish women aged 20–40. This overrepresentation may have raised the average score by 1–2 points. Additionally, knowledge was assessed using a non-validated 15-item tool, which may have introduced measurement error. Although internal consistency measures (e.g., Cronbach's α) were not applicable due to the factual, non-latent nature of the knowledge items, the questionnaire underwent expert review and pilot testing to ensure content validity and clarity. As all data were self-reported and cross-sectional, causality cannot be inferred—for instance, whether private care increases knowledge or whether more informed women choose private providers. Finally, the survey did not assess behaviors or serostatus, limiting interpretation of knowledge–action links. Nonetheless, the study benefits from a large national sample, rich socio-economic and healthcare data, and separate reporting by parity—an uncommon feature in CMV research. (Greye et al., 2022; Pereboom et al., 2013)

## 5 Conclusions

This nationwide survey shows that pregnant women in Poland have only moderate knowledge about CMV, with notable socio-demographic and care-related disparities. Urban residence, higher education, and private antenatal care were linked to greater awareness, while parity showed no association, possibly reflecting inconsistent counselling. These findings highlight the need for structured CMV education integrated into routine public care and for professional training to ensure consistent prevention messaging. Future studies should examine how maternal knowledge translates into preventive behaviors and infection outcomes.

## CRediT authorship contribution statement

**Magda Rybak-Krzyszkowska:** Writing – original draft, Methodology, Investigation, Conceptualization. **Michał Strus:** Writing – review & editing, Software, Investigation, Data curation. **Hubert Huras:** Writing – review & editing, Validation, Supervision, Resources. **Wojciech Górczewski:** Writing – review & editing, Visualization, Investigation. **Maciej W. Socha:** Writing – review & editing, Data curation. **Lidia Stopyra:** Writing – review & editing, Investigation, Conceptualization. **Dorota Sys:** Writing – original draft, Methodology, Investigation, Formal analysis, Conceptualization.

## Ethics approval and consent to participate

The study was conducted in accordance with the ethical principles of medical research, including the Declaration of Helsinki. Only fully anonymized data were collected and analyzed. This research does not meet the definition of a medical experiment as per Article 21 of the Act on the Profession of Physician of 5 December 1996 (consolidated text: Journal of Laws 2021, item 790), nor the definition of a clinical trial under Article 2 (2) (2) of Regulation (EU) 536/2014, as implemented by the Act on Clinical Trials of Medicinal Products Used in Humans of 9 March 2023. Therefore, under applicable Polish law, no ethics committee approval was required. Participation was entirely anonymous and voluntary. Completion and submission of the online questionnaire constituted informed consent to participate in the study.

## Declaration of generative AI and AI-assisted Technologies in the Writing Process

During the preparation of this manuscript, the authors used ChatGPT (OpenAI) to improve the readability and language of the text. After generating suggestions with this tool, the authors reviewed, edited, and approved all content and take full responsibility for the accuracy and integrity of the final manuscript.

## Funding

This research did not receive any specific grant from funding agencies in the public, commercial, or not-for-profit sectors.

## Declaration of competing interest

The authors declare that they have no known competing financial interests or personal relationships that could have appeared to influence the work reported in this paper.

## Data Availability

Data will be made available on request.
